# Linalyl acetate restores colon contractility and blood pressure in repeatedly stressed-ulcerative colitis rats

**DOI:** 10.1265/ehpm.22-00041

**Published:** 2022-06-24

**Authors:** You Kyoung Shin, Soonho Kwon, Yu Shan Hsieh, A Young Han, Geun Hee Seol

**Affiliations:** 1 Department of Basic Nursing Science, School of Nursing, Korea University, Seoul, Republic of Korea; 2 Department of Nursing, School of Nursing, National Taipei University of Nursing and Health Sciences, Taipei, Taiwan; 3 Department of Nursing, School of Nursing, Sunchon National University, Sunchon, Republic of Korea; 4 BK21 FOUR Program of Transdisciplinary Major in Learning Health Systems, Graduate School, Korea University, Seoul, Republic of Korea

**Keywords:** Repeated stress, Ulcerative colitis, Linalyl acetate, Colon contractility, Blood pressure

## Abstract

**Background:**

Ulcerative colitis (UC) is related to stress, but few studies have evaluated the influence of stress on factors affecting colon contractility in rats with UC. Also, there have been no studies investigating beneficial effects of linalyl acetate (LA), the major component of lavender essential oil, in repeatedly stressed-ulcerative colitis rats. Therefore, we investigated the differences in factors affecting colon contractility of UC rats with or without repeated restraint stress (RRS) and the effects of LA on these parameters in repeatedly stressed-UC rats.

**Methods:**

Rats were assigned to following groups: control, RRS, UC, RRS+UC, and RRS+UC treated with LA or sulfasalazine. To induce UC, rats were administered 2% dextran sodium sulfate (DSS) water on days 1–5, followed by tap water on days 6–15 and DSS water on days 16–20. RRS was induced by immobilizing rats for 2 hr/day on days 1–20. LA or sulfasalazine were daily administered on days 16–20.

**Results:**

Disease activity index (DAI) was markedly increased in RRS+UC. Serum interleukin-6 levels and acetylcholine-induced colon contraction were higher in RRS+UC than in control, RRS and UC. Colon nitrite levels also significantly increased in RRS+UC compared to the control and RRS. Blood pressure (BP) was higher in RRS+UC than in the control and UC. Both LA and sulfasalazine was effective in decreasing DAI, colon nitrite levels, acetylcholine-induced colon contraction in RRS+UC. Sulfasalazine significantly reduced serum IL-6 levels in RRS+UC with decreasing tendency in RRS+UC treated by LA. Only LA significantly reduced BP in RRS+UC.

**Conclusions:**

Our findings emphasize the importance of stress management in UC patients. Also, LA may be beneficially used in repeatedly stressed-UC patients with high BP.

## Background

In modern society, stress has become a significant problem that induces negative health outcomes via neuroendocrine and autonomic responses and changes in health behaviors [1]. Epidemiological studies indicate that exposure to environmental factors such as stress is related to an increased risk of many human illnesses, including cardiovascular diseases and neuronal dysfunction [2, 3].

Ulcerative colitis (UC) is an inflammatory bowel disease (IBD) that has worldwide impacts and exhibits increased incidence in newly industrialized countries [4]. Accumulated clinical findings have shown that disease activity in UC patients is related to stress. For example, perceived stress has been strongly associated with disease activity and health-related quality of life in UC patients [5]. Also, various studies have examined how stress affects the pathogenesis of UC in rodent models of the disease. Acute 4 hr stress after colitis induction was found to change the immune response in mice [6], and restraint stress for 7 days along with colitis induction accelerated colitis by impairing gut microbiota and damaging the mucus layer [7]. Although consideration of stress may be an important strategy for preventing and managing UC, no study to date has investigated the effect of concurrent repeated stress in a rodent model of UC with relapse and remission periods.

Previous studies reported that stress modulates intestinal movement [8] and affects the altered gut motility seen in IBD [9]. IBD can also exhibit cardiovascular manifestations, which may occur concomitantly with gastrointestinal manifestations [10]. Patients with IBD showed significantly increased carotid intima-media thickness and carotid-femoral pulse wave velocity compared to controls, suggesting that these patients had subclinical atherosclerosis [11]. Patients with UC have also been suggested to have an elevated risk of ischemic heart disease [12]. To date, however, no published study has examined the influence of repeated stress on factors affecting colon contractility in a rodent model of UC.

Linalyl acetate (LA), the main components of lavender essential oil, has been reported to have anti-inflammatory effects in chronic obstructive pulmonary disease (COPD)-like and hypertensive rats [13] and to reduce serum corticosterone levels in stressed-diabetic rats [14]. These findings suggest that LA may have beneficial effects on repeatedly stressed-UC rats, but no studies have explored the effects of LA in repeatedly stressed-UC rats. Accordingly, we aimed to investigate the differences in factors affecting colon contractility of UC rats with or without repeated restraint stress (RRS) exposure, and to identify the effects of LA in repeatedly stressed-UC rats.

## Methods

### Experimental design

All experimental procedures were approved by the institutional animal research and ethics committee of Korea University (KUIACUC-2016-153). Sprague-Dawley rats (male, 4 weeks of age) were housed under a 12-hr dark-light cycle at 22–23 °C with free access to regular chow and tap water. Forty-five rats were randomly assigned to the following groups: control (n = 6), RRS (n = 6), UC (n = 7), RRS+UC (n = 7), and RRS+UC rats treated with 10 mg/kg LA (RRS+UC+LA10, n = 5), 100 mg/kg LA (RRS+UC+LA100, n = 7), or 200 mg/kg sulfasalazine (RRS+UC+Sulfasalazine, n = 7).

Considering that UC is a chronic disease with varying periods of relapse and remission, the induction of chronic UC was achieved as previously described. Briefly, rats were given free access to autoclaved drinking water in which 2% dextran sodium sulfate (DSS) solution had been dissolved. DSS-replaced water was provided on days 1–5 (acute phase). On days 6–15, tap water (without DSS) was provided for recovery phase. After that, rats were provided with DSS-replaced water on days 16–20 (chronic phase) [15]. Rats in the control and RRS groups were freely allowed to drink autoclaved tap water on days 1–20. RRS was induced according to previous study showing that exposure to RRS for 2 hr/day during 20 days induced anxiety-like behavior representing psychological stress conditions [16]. Briefly, rats of the RRS and RRS+UC groups were immobilized in an adjustable cylinder for 2 hr/day on days 1–20, whereas rats in the control and the UC were allowed to rest freely in their cages.

**Fig. 1:**
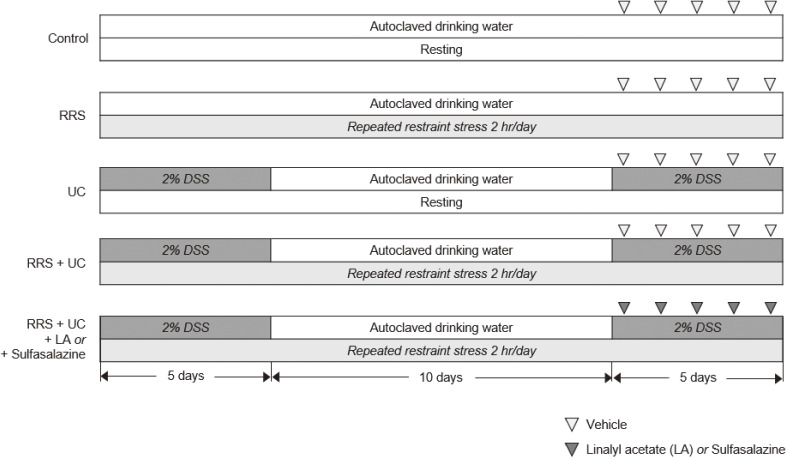
Experimental design. DSS, dextran sodium sulfate; LA, linalyl acetate; RRS, restraint stress; UC, ulcerative colitis

Rats in the LA10 and LA100 groups were intraperitoneally administered LA, diluted in propylene glycol once daily on days 16–20, whereas rats in the Sulfasalazine group were orally administered sulfasalazine, diluted in normal saline, daily on days 16–20. The concentrations of LA [13] and sulfasalazine [17] were based on the results of previous studies. Before measuring colon contractility, rats were fasted for 24 hr [18], and they were anesthetized with isoflurane and decapitated on day 21. Blood and tissue samples were collected for further evaluation (Fig. 1).

### Disease activity index (DAI)

DAI was calculated from the parameters of stool consistency, weight loss, and perianal bleeding, as previously described [6]. Higher scores indicated increased UC activity.

### Blood pressure (BP)

Systolic and diastolic BP non-invasively measured using a tail cuff transducer (Kent Scientific, Torrington, USA). BP was measured at least 5 times per rat at each time point and the mean BP was calculated. Systolic and diastolic BP were assessed 30 min after restraint stress on day 1, 6, 11, 16 and 20. Rats were placed in an animal holder and stabilized on a warming platform before the measurement.

### Colon contraction

The colons were dissected and rapidly immersed in physiologic solution. The inner contents were carefully washed away, the colon samples were cut into 15–20 mm pieces, which were mounted on a mechanotransducer (Danish Myo Technology, Aarhus, Denmark). Each organ bath was continuously oxygenated and maintained at 37 °C. After each sample was stabilized at an optimal baseline tension of 0.5–1.0 g for 1 hr, contraction was induced by adding 1 µM acetylcholine (ACh).

### Serum interleukin (IL)-6

Serum concentrations of IL-6 was measured using rat IL-6 (KomaBio, Seoul, Korea) ELISA kits. Results were obtained by measuring absorbance at 450 nm using a microplate ELISA reader according to the manufacturer’s instructions.

### Colon tissue nitrite

Colon tissue was homogenized and lysed for 30 min, and the lysate was centrifuged for 10 min at 13,000 g. Nitrite concentrations in lysate were determined by adding sulfanilamide hydrochloride and N-(1-naphthyl) ethylenediamine dihydrochloride and measuring absorbance at 540 nm using a microplate ELISA reader.

### Chemicals

DSS was purchased from MP Biomedicals (OH, USA), and all other chemicals used in this study were purchased from Sigma-Aldrich (MO, USA).

### Statistical analysis

Data were presented as mean ± SEM and analyzed using the SPSS version 22 (IL, USA). DAI data were analyzed using repeated measures analysis of variance (ANOVA), which revealed a significant interaction among groups and time (days) for DAI. Therefore, the effects of LA on DAI were tested by one-way ANOVA using data from day 20 followed by a post-hoc LSD test. BP data were analyzed using one-way ANOVA at each time point (day 1, 6, 11, 16 and 20) followed by a post-hoc LSD test, and all other variables were analyzed using one-way ANOVA followed by post-hoc comparisons with LSD tests. *P* < 0.05 was considered statistically significant.

## Results

**Fig. 2:**
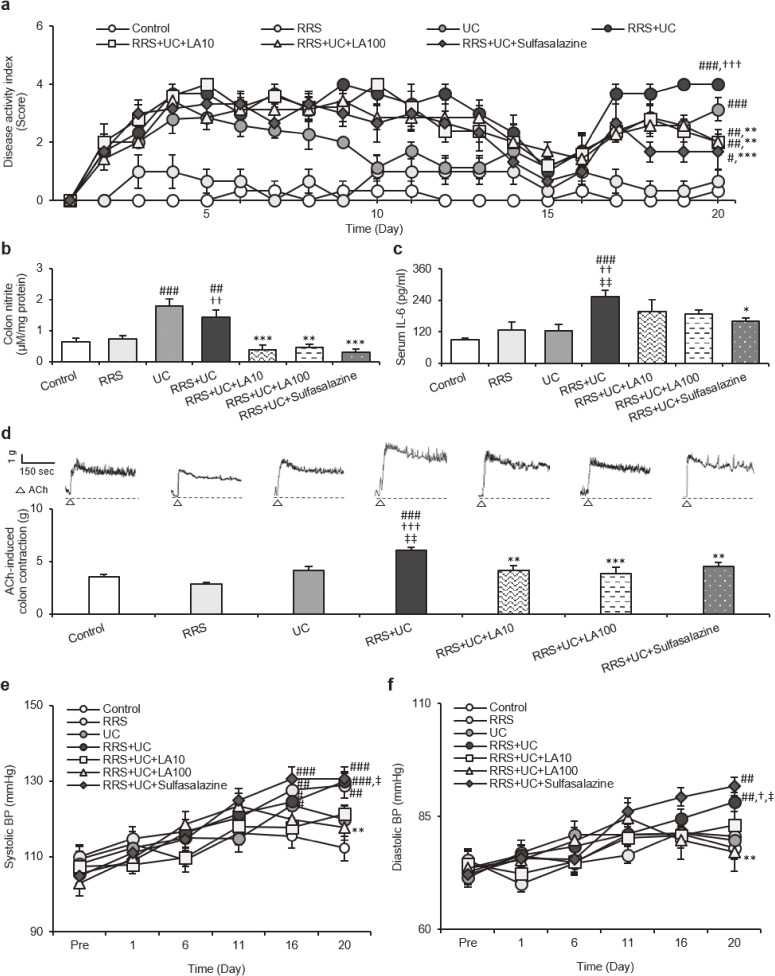
Effects of LA on factors affecting colon contractility and BP in repeatedly stressed-UC rats. **(a)** Disease activity index. **(b)** Nitrite levels in colon tissue. **(c)** Serum IL-6 levels. **(d)** Acetylcholine-induced colon contraction. **(e)** Systolic BP. **(f)** Diastolic BP. Results are presented as mean ± SEM. DAI data were analyzed by repeated measures ANOVA and one-way ANOVA using data from day 20. BP data were analyzed using one-way ANOVA at each time point followed by a post-hoc LSD test. ^#^*P* < 0.05, ^##^*P* < 0.01, ^###^*P* < 0.001 vs. Control; ^†^*P* < 0.05, ^††^*P* < 0.01, ^†††^*P* < 0.001 vs. RRS; ^‡^*P* < 0.05, ^‡‡^*P* < 0.01 vs. UC; *P < 0.05, **P < 0.01, ***P < 0.001 vs. RRS+UC. ACh, acetylcholine; BP, blood pressure; IL, interleukin; LA, linalyl acetate; RRS, restraint stress; UC, ulcerative colitis

On day 20, DAI increased significantly in order of the control, UC (*P* < 0.001) and RRS+UC (*P* < 0.001) groups. LA 10 mg/kg (*P* = 0.002), LA 100 mg/kg (*P* = 0.001), and sulfasalazine (*P* < 0.001) significantly reduced DAI in the RRS+UC group (Fig. 2a).

Nitrite levels in colon tissue were significantly higher in the UC (*P* < 0.001) and RRS+UC (*P* = 0.001) groups than in the control group. LA 10 mg/kg (*P* < 0.001), LA 100 mg/kg (*P* = 0.001), and sulfasalazine (*P* < 0.001) significantly reduced colon nitrite in the RRS+UC group (Fig. 2b). Serum IL-6 concentrations were significantly higher in the RSS+UC group than in the control (*P* < 0.001), RRS (*P* = 0.001) and UC (*P* = 0.001) groups, with serum IL-6 concentrations tending to be higher in the Stress and UC groups than in the control group. Rats in the RRS+UC group treated with sulfasalazine significantly decreased serum IL-6 concentrations (*P* = 0.035). Treatment of LA 100 mg/kg showed decreasing tendency of serum IL-6 levels (*P* = 0.066) although there were no significant differences (Fig. 2c). Although the differences were not statistically significant, ACh-induced colon contraction was higher in the UC than in the control. In addition, ACh-induced colon contraction was markedly higher in the RRS+UC group than in the control (*P* < 0.001), RRS (*P* < 0.001) and UC (*P* = 0.001) groups. LA 10 mg/kg (*P* = 0.004), LA 100 mg/kg (*P* < 0.001), and sulfasalazine (*P* = 0.003) significantly reduced ACh-induced colon contraction in the RRS+UC group, with ACh-induced colon contraction restored to the control levels by LA 100 mg/kg (Fig. 2d).

For both systolic and diastolic BP, no significant differences were observed among data from days 1, 6 and 11. On day 16 when LA treatment was started, systolic BP was significantly increased in the RRS (*P* = 0.006), UC (*P* = 0.039), RRS+UC (*P* = 0.036) and RRS+UC+Sulfasalazine (*P* < 0.001) groups compared to the control group. Both systolic and diastolic BP showed a decreasing tendency in the LA-treated groups compared with the RRS+UC group, but there was no significant difference. On day 20, Systolic BP was significantly higher in the RRS group than that of the control (*P* = 0.001). Also, systolic BP was significantly higher in the RRS+UC group than that of the control (*P* < 0.001) and UC groups (*P* = 0.020). On day 16, diastolic BP showed a tendency to increase in the RRS+UC and RRS+UC+Sulfasalazine groups compared to the control group, but statistical significance was not reached (*P* = 0.966, *P* = 0.277 respectively). Similar to systolic BP, on day 20 diastolic BP was significantly higher in the RRS+UC than in the control (*P* = 0.008), RRS (*P* = 0.046) and UC groups (*P* = 0.026). Systolic and diastolic BP were significantly reduced only by the LA 100 mg/kg (Fig. 2e, 2f).

## Discussion

In this study, we investigated the effects of RRS on DSS-induced chronic UC rats, with a particular focus on factors affecting colon contractility. The RRS+UC group exhibited a slower decrease in DAI compared to the UC group during the remission period (from day 6 to day 15), and the RRS+UC group showed a faster increase in DAI compared to the UC group during the relapse period (from day 16 to day 20). In accordance with our results, stressful life events have been found to increase the risk of relapse in UC patients during remission periods [19]. Similarly, a previous clinical study reported that psychological distress levels were significantly associated with the total number of relapses in patients with inactive IBD [20]. It is well known that stress induces the production of catecholamines. For example, circulating inflammatory cytokines were increased in rats exposed to tail-shock stress, suggesting that catecholamines are important mediators of stress [21]. Moreover, resistance to glucocorticoids occurs during stress, leading to high plasma concentrations of proinflammatory cytokines such as IL-6 [22]. Although nitric oxide (NO) generated by constitutive nitric oxide synthase (NOS) controls physiological conditions in the colon, high levels of NO generated by inducible NOS contribute to inflammatory responses [23]. These findings support our results, showing that, compared with the control group, IL-6 tended to be higher in the RRS and UC groups and significantly higher in the RRS+UC group. Finally, intestinal inflammation has been identified as a stronger predictor of symptom activity in UC patients [24]. Collectively, these lines of evidence indicate that RRS may exacerbates UC in this study.

Interestingly, ACh-induced colon contraction was significantly increased in the RRS+UC group compared to the control, RRS and UC groups. It had been reported that stress increased ACh-induced colon contraction compared to controls [8]. Also, corticotropin-releasing factor (the main activator of the hypothalamic-pituitary-adrenal axis under stress) increased colonic motility and this effect could be inhibited by atropine pre-treatment, suggesting that cholinergic signaling is involved through muscarinic receptors [25]. Corticotropin-releasing factor also stimulated propulsive colonic motor function and diarrhea in rats [26]. Altered colonic motility may contribute to increased frequency and urgency of defecation in UC [27]. Because perceived stress was associated with disease activity in UC patients [5], we investigated ACh-induced colon contraction of UC rats with or without RRS. Therefore, our results suggest that the colon is susceptible to RRS in UC, and indicate that the increased DAI score in the RRS+UC group may be related to hypercontraction of the colon occurring due to upregulated muscarinic signaling.

In present study, the systolic and diastolic BP, indicators that reflect the level of stress in our study, were significantly increased in the RRS+UC group compared to the control and UC groups. Patients with IBD have reported various type of life stressors such as family, work, and financial stress [28], and in a secondary analysis, hypertension was more frequently diagnosed after than before diagnosis of IBD [29]. A previous study showed that chronic stress increased circulating catecholamines by activating the sympathetic nervous system and hypothalamic-pituitary-adrenal axis in mice [30]. It is well recognized that catecholamines elevate BP. Acute psychological stress increased systolic BP by 12 mmHg in UC patients, whereas same stressor increased systolic BP by 9 mmHg in healthy participants [31]. Moreover, in that study, acute psychological stress increased diastolic blood pressure only in UC patients. Within the context of these previous reports, our findings indicate that RRS abnormally increased the BP of UC rats. Therefore, it may be important to assess cardiovascular parameters to detect cardiovascular manifestations in UC patients with repeated stress.

Because there is no effective treatment for UC, with current treatments only relieving its symptoms, natural products with antiulcerogenic activities are considered potential sources of UC treatments [32]. Therefore, we investigated the effects of LA, the major component of lavender essential oil, in repeatedly stressed-UC rats. In summary, we found that LA was effective in reducing DAI and colon nitrite in the RRS+UC group at both concentrations. The cause of the dose-independent effect of LA on colon nitrite in the RRS+UC group is not known, but the abovementioned previous study also found that 10 mg/kg LA was a little bit more potent than 100 mg/kg LA in normalizing serum nitrite levels in COPD-like hypertensive rats [13]. There are various factors that affect DAI, including colon nitrite levels, and the sum of the effects of LA on these factors may result in a dose-independent effect of LA on DAI in the RRS+UC group. Also, we found that LA was effectively reduced ACh-induced colon contraction in repeatedly stressed-UC rats, similar to the effect of sulfasalazine, a drug used in the conventional treatment of UC. Although sulfasalazine was effective in lowering serum-IL6 levels in repeatedly stressed-UC rats, LA also showed obvious tendency to reduce serum IL-6. In addition, only LA reduced elevated BP in repeatedly-stressed UC rats.

In previous research, LA was shown to prevent inflammatory responses by reducing pro-inflammatory cytokines in bronchoalveolar lavage fluid from COPD-like and hypertensive rats [13]. LA was reported to inhibit olmesartan-induced intestinal hypermotility in hypertensive rats [33]. Also, LA was shown to reverse acute nicotine-induced cardiovascular changes in rats [34]. LA contains a hydroxyl group [35]. The hydroxylated compounds exhibited higher potency in the relaxation of rat aortas pre-contracted with BayK-8644 than did limonene, which does not have a hydroxyl group, indicating that the presence of a hydroxyl group was associated with pharmacological potency on vasorelaxation [36]. Also, direct vasorelaxant effects of LA [37] may be related to the reduced BP in repeatedly stressed-UC rats. Determination of more specific mechanisms requires additional studies to evaluate the effects of LA on other pro-inflammatory cytokines, pathological features and levels of antioxidant enzymes in repeatedly stressed-UC rats.

## Conclusion

Our findings provide directions for future clinical studies on the value of assessing and managing stress to ameliorate UC. To our knowledge, this is the first report on the effects of LA in repeatedly stressed-UC rats. LA may be suitable for reducing both disease activity due to hypercontraction of the colon and BP in repeatedly stressed-UC patients with high BP.

## Abbreviations

ACh: Acetylcholine
DAI: Disease activity index
DSS: Dextran sodium sulfate
IBD: Inflammatory bowel disease
IL: Interleukin
LA: Linalyl acetate
NO: Nitric oxide
NOS: Nitric oxide synthase
RRS: Repeated restraint stress
UC: Ulcerative colitis

## Declarations

### Availability of data and material

Data are available on request from corresponding author.
